# A remote management system for control and surveillance of echinococcosis: design and implementation based on internet of things

**DOI:** 10.1186/s40249-021-00833-4

**Published:** 2021-04-13

**Authors:** Shi-Jie Yang, Ning Xiao, Jing-Zhong Li, Yu Feng, Jun-Ying Ma, Gong-Sang Quzhen, Qing Yu, Ting Zhang, Shi-Cheng Yi, Xiao-Nong Zhou

**Affiliations:** 1 National Institute of Parasitic Diseases, Chinese Center for Disease Control and Prevention, Shanghai, China; 2 NHC Key Laboratory of Parasite and Vector Biology, (National Institute of Parasitic Diseases, Chinese Center for Disease Control and Prevention), Shanghai, China; 3 National Center for International Research on Tropical Diseases, Shanghai, China; 4 WHO Collaborating Center for Tropical Diseases, Shanghai, China; 5 School of Global Health, Chinese Center for Tropical Diseases Research, Shanghai Jiao Tong University School of Medicine, Shanghai, China; 6 Tibet Center for Disease Control and Prevention, NHC Key Laboratory of Echinococcosis Prevention and Control, Lhasa, China; 7 Department of Parasitic Diseases, Gansu Center for Disease Control and Prevention, Lanzhou, China; 8 Qinghai Institute for Endemic Disease Prevention and Control, Xining, China; 9 Shanghai Yier Information Technology Co., Ltd, Shanghai, China

**Keywords:** Remote Management System, Control, Surveillance, Echinococcosis, Design, Implementation, Internet of Things

## Abstract

**Background:**

As a neglected cross-species parasitic disease transmitted between canines and livestock, echinococcosis remains a global public health concern with a heavy disease burden. In China, especially in the epidemic pastoral communities on the Qinghai-Tibet Plateau, the harsh climate, low socio-economic status, poor overall hygiene, and remote and insufficient access to all owned dogs exacerbate the difficulty in implementing the ambitious control programme for echinococcosis. We aimed to design and implement a remote management system (RMS) based on internet of things (IoT) for control and surveillance of echinococcosis by combining deworming devices to realise long-distance smart deworming control, smooth statistical analysis and result display. New methods and tools are urgently needed to increase the deworming coverage and frequency, promote real-time scientific surveillance, and prevent transmission of echinococcosis in remoted transmission areas.

**Methods:**

From 2016 to 2019, we had cooperated and developed the smart collar and smart feeder with the Central Research Institute of Shanghai Electric Group Co., Ltd. (Shanghai, China) and Shenzhen Jizhi Future Technology Co., Ltd. (Shenzhen, China). From September 2019 to March 2020, We had proposed the RMS based on IoT as a novel tool to control smart deworming devices to deliver efficient praziquantel (PZQ) baits to dogs regularly and automatically and also as a smart digital management platform to monitor, analyse, and display the epidemic trends of echinococcosis dynamically, in real time in Hezuo City, Gannan Tibetan Autonomous Prefecture, Gansu Province, China. Starting from January 2018, The RMS has been maintained and upgraded by Shanghai Yier Information Technology Co., Ltd (Shanghai, China). The database was based on MySQL tools and the Chi-square test was used to probe the difference and changes of variables in different groups.

**Results:**

The smart collars are fully capable of anti-collision, waterproof, and cold-proof performance, and the battery’s energy is sufficient, the anti-collision rate, water-proof rate, cold-proof rate and voltage normal rate is 99.6% (521/523), 100.0% (523/523), 100.0% (523/523) and 100.0% (523/523), respectively. The RMS can accurately analyse the monitoring data and parameters including positive rates of canine faeces, and the prevalence of echinococcosis in the general population livestock, and children. The data of dogs deworming and surveillance for echinococcosis is able to be controlled using RMS and has expanded gradually in townships to the whole Hezuo region. The automatic delivering PZQ rate, collar positioning rate, deliver PZQ reminding rate, and fault report rate is 91.1% (1914/2102), 92.1% (13 580/14 745), 92.1% (1936/2102) and 84.7% (1287/1519), respectively. After using the RMS from 2019, the missing rate of monitoring data decreased from 32.1% (9/28) to 0 (0/16). A total of 48 administrators (3, 3, 8, 11, 23 at the provincial, municipal, county, township, village levels, respectively) participated in the questionnaire survey, with 93.8% of its overall satisfaction rate.

**Conclusions:**

The existing difficulties and challenges in the way of prevention and control for echinococcosis can partially be resolved using the innovative, IoT-based technologies and tools. The proposed RMS advance the upgrade of existing manual prevention and control models for echinococcosis, especially in the current ongoing COVID-19 pandemic, as social distance and community blockade continue.

**Graphic abstract:**

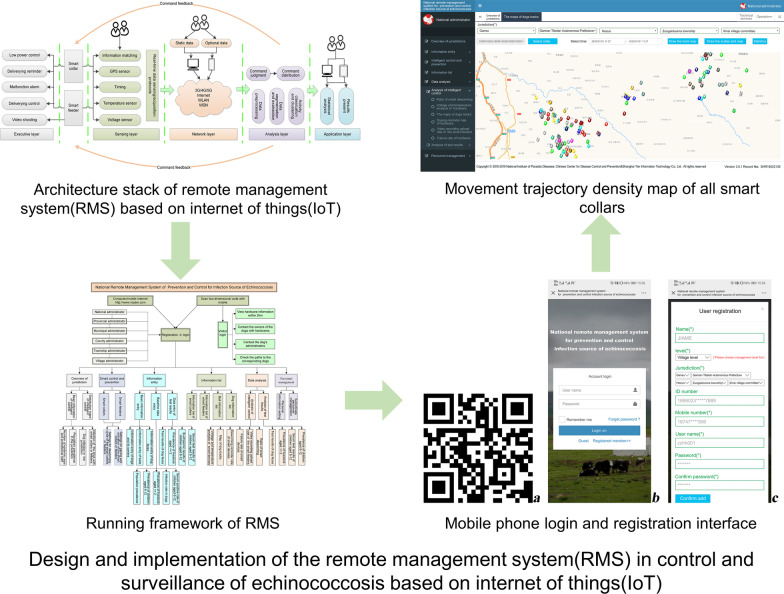

**Supplementary Information:**

The online version contains supplementary material available at 10.1186/s40249-021-00833-4.

## Background

Echinococcosis, a zoonotic disease characterised by cross-species spillover from canines to humans, livestock, and rodents, is a serious public health concern. In 2010, the World Health Organization (WHO) listed echinococcosis as a neglected zoonotic disease and advocated for preferential control [1, 2]. Dogs are the definitive hosts of the causative parasites, *Echinococcus granulosus* and *E. multilocularis*. In this regard, the WHO and the World Organization for Animal Health (OIE) recommended periodic deworming for dogs to stop disease transmission [3]. However, manual deworming faces significant challenges especially in resource-poor remote areas due to the high altitude, harsh climate, unique religious and cultural customs, the poor infrastructure, unavailable veterinary human resources, nomadic lifestyles, low socio-economic status, and overall poor hygiene conditions[4]. The existing labour-intensive deworming encompasses delivery of the praziquantel (PZQ) baits to the dog owners, who then feed the dogs once a month, or a local veterinarian is assigned to visit the owner’s house once a month to administer the PZQ bait to each dog [3, 5]. The deworming coverage and frequency for dogs are far lower than those recommended by the WHO/OIE, i.e. ‘4–8 times per year’ and ‘at least > 90% of registered dogs’ [3, 5–7] leading to the relatively high prevalence of *Echinococcus* spp. among dogs in China, i.e. 2.96% in Sichuan, 3.03% in Ningxia, 4.91% in Gansu, 7.3% in Tibet Autonomous Region (TAR), 13.0% in Qinghai, and 41.3% in Hobukesar Mongol Autonomous County in northwest Xinjiang [4, 7, 8]. The underlying reasons for the high prevalence of *Echinococcus* spp. in dogs include the ubiquitous nature of hosts coupled by a heavy workload against few staff. The local Centre for Disease Control and Prevention (CDC) and the veterinary station store surveillance data in spreadsheets or paper materials that are usually incomplete, inaccurate, and untimely, making it difficult to combine, analyse, and utilise the data in a proper way.

The new wave of technological advancements has provided an opportunity to overcome these limitations. As an innovative technology, the internet of things (IoT), is increasingly being used across various sectors, such as industry, agriculture, transportation, banking, government, and healthcare system [9]. By 2018, there were approximately 13 medical sub-fields associated with medical IoT, and 79% of medical IoT applications were focused on neurology, cardiology, and psychiatry/psychology. However, a recent systematic review revealed that only 3.4% (3/89) of all the evaluated IoT articles involved the monitoring and control of infectious diseases [9]. Sareen et al. established a Naive Bayesian Network (a cloud-based system) to help the government control the spread of Zika virus disease [10]. A novel architecture based on radio frequency identification device, wearable sensor technology, and cloud computing infrastructure is proposed for the monitoring of Ebola-infected patients [11]. IoT-assisted fog health monitoring system was used to identify possibly people infected by the Chikungunya virus in an early phase of their illness [12]. In particular, web-based tools (Google Flu Trends) for surveillance of the flu virus have been utilised to monitor, in real time, influenza activity [13]. In the current COVID-19 pandemic, IoT is expected to be useful in simultaneous monitoring, surveillance, early warning, prevention, and treatment [14–16]. In this study, we aimed to design and implement a remote management system (RMS) for control and surveillance of echinococcosis by combining deworming devices, sensors, information technology, and available dynamic networking devices. The RMS is expected to realise long-distance deworming control, smooth statistical analysis and result display, and ultimately improve the efficiency and quality of control and surveillance of echinococcosis.

## Methods

### Facilities

We cooperated and developed the smart collar and smart feeder with the Central Research Institute of Shanghai Electric Group Co., Ltd. (Shanghai, China) and Shenzhen Jizhi Future Technology Co., Ltd. (Shenzhen, China). The temperature sensor, GPS sensor, voltage sensor, low-power technology, and timing were employed to ensure the realisation of a series of functions [17, 18]. The required executive modules of PZQ-delivery, malfunction alarm, video shooting, low-power control, and delivery reminder were all embedded and controlled by the central processing unit (CPU) [19]. The smart devices are fully equipped with waterproof, anti-collision, and cold-proof capabilities.

Wireless networking technology is employed to ensure data exchange command [20]. The integrated transmission control protocol (TCP)/internet protocol supports Wi-Fi access by the microcontroller access [21]. China Mobile Network is used for communication of data and commands between smart devices, RMS, and administrators. The 3rd, 4th, and 5th generation mobile communication (3G/4G/5G) have been adopted according to the network assembly situation and locality.

### Database and server

Our database was based on MySQL tools as it is the most widely used Relational Database Management System [22]. The core programme adopts the entire multi-threaded programming and supports the formal structured query language and various data types to perform detailed queries. The static, optional, and real-time data lists are created subsequently (Table 1).

**Table 1:** Classification of datasets for remote management system (RMS)

Classification of datasets	Attributes and contents	Administrators
Static data	Names of the village, codes, geographic locations (latitude and longitude), basic hardware attributes, physical properties, and manufacturers, batch number, basic parameters in each township of 368 counties and 73 cities (prefectures) in 9 echinococcosis epidemic provinces (regions), etc	National administrators
Optional data	Hardware ID, information of PZQ baits	Provincial/municipal/county administrators
	Information of owner, village administrator, and dog;	Village administrators
	Baseline information of echinococcosis epidemic in 2012–2016; etc	Provincial/municipal/county administrators
Real-time data	Temperature and power information of smart collars and feeders; dog's location; information of deworming, reminder, alarm, video recording, and uploading, etc	Updating by RMS automatically
	Basic data such as dog faeces information children's information, Serum information, intermediate host information	Township/village’s administrators
	Test results of dog faeces, children's serum; ultrasound screening results; test results of the intermediate host, etc	Provincial/municipal/county administrators
	Matching the information of dogs, owners, smart collars or feeders, and township/village’ s administrators, etc	Township/village administrators
	Other inputting information and command to be executed and matched, e.g. adjusting the time, period and dosage of delivering PZQ baits, etc	Provincial/municipal administrators
	Inputting or activation of the information of administrator	Provincial/municipal/county/township administrators

We used .NET development environment as our web server and the HTML-embedded language to create a dynamic interactive website with B/S framework [23].

### Field settings

From September 2019 to March 2020, a total of 523 smart collars were attached to the dogs for smart deworming in the pastoral area of Hezuo, Gannan Tibetan Autonomous Prefecture, Gansu Province, where the total population of residents is about 100 000, of which 55% are ethnic Tibetan (http://www.hezuo.gov.cn/info/1178/22963.htm), and the livestock population is around 291 300 head (https://baike.baidu.com/item/%E5%90%88%E4%BD%9C/2640496?fromtitle=%E5%90%88%E4%BD%9C%E5%B8%82&fromid=10794996&fr=aladdin#reference-[1]-5063862-wrap). The prevalence of echinococcosis was 0.13% in the human population, 3.18% among dogs (a total of 7463 dogs were registered), and 3.43% among livestock [24]. The RMS was installed in Hezuo Centre for Disease Control and Prevention. The questionnaire survey on satisfaction of RMS was implemented at the five levels of administrators. The canine faecal samples were collected once a month from both groups, and the tests were conducted once a quarter in Hezuo Center for Disease Control and Prevention, Gansu province, China. The coproantigen ELISA Kit for Canine was produced by Shenzhen Combined Biotech Co., Ltd., Shenzhen, China. To ensure quality, the testing staff were trained using the same batch of testing kits. The operation steps were followed as per the product manual.

### Data analysis

The maps and boundaries of the villages, townships, counties, cities, and provinces have been obtained from the National Surveying and Mapping Geographic Information Bureau (http://www.webmap.cn/main.do?method=index) and GaodeMaps (https://www.amap.com/). The names and codes for the administrative divisions of the People's Republic of China are from the website of the National Bureau of Statistics (http://www.stats.gov.cn/tjsj/tjcbw/201512/t20151210_1287837.html), and the basic smart devices attribute information, and the basic information of PZQ bait are provided by the manufacturer (Shanghai Yier Information Technology Co., Ltd, Shanghai, China). The number of dogs in each of 368 counties is from the National Echinococcosis Prevention and Treatment Report (2016, unpublished). The baseline data is derived from the National Report for Hydatidosis Epidemiological Survey [25, 26]. We used SPSS statistical software version 20 (IBM Corp, Armonk, NY, USA) for data analysis. The Chi-square test was used to probe the difference and changes of the canine faecal antigen. The statistical significance difference was set to *P* < 0.05.

## Results

### Architecture of RMS

**Fig. 1:**
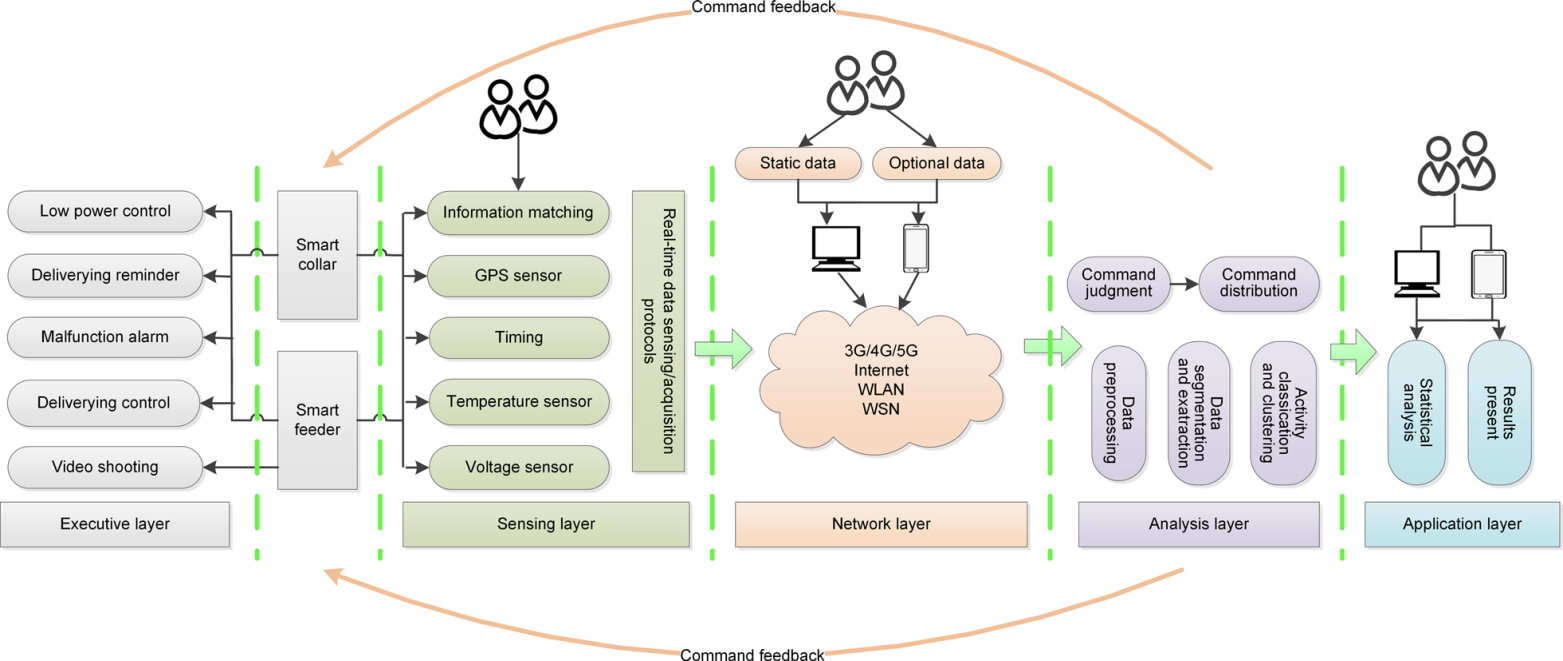
Architecture stack of RMS base on IoT. RMS: Remote Management System; IoT: Internet of Things

**Fig. 2:**
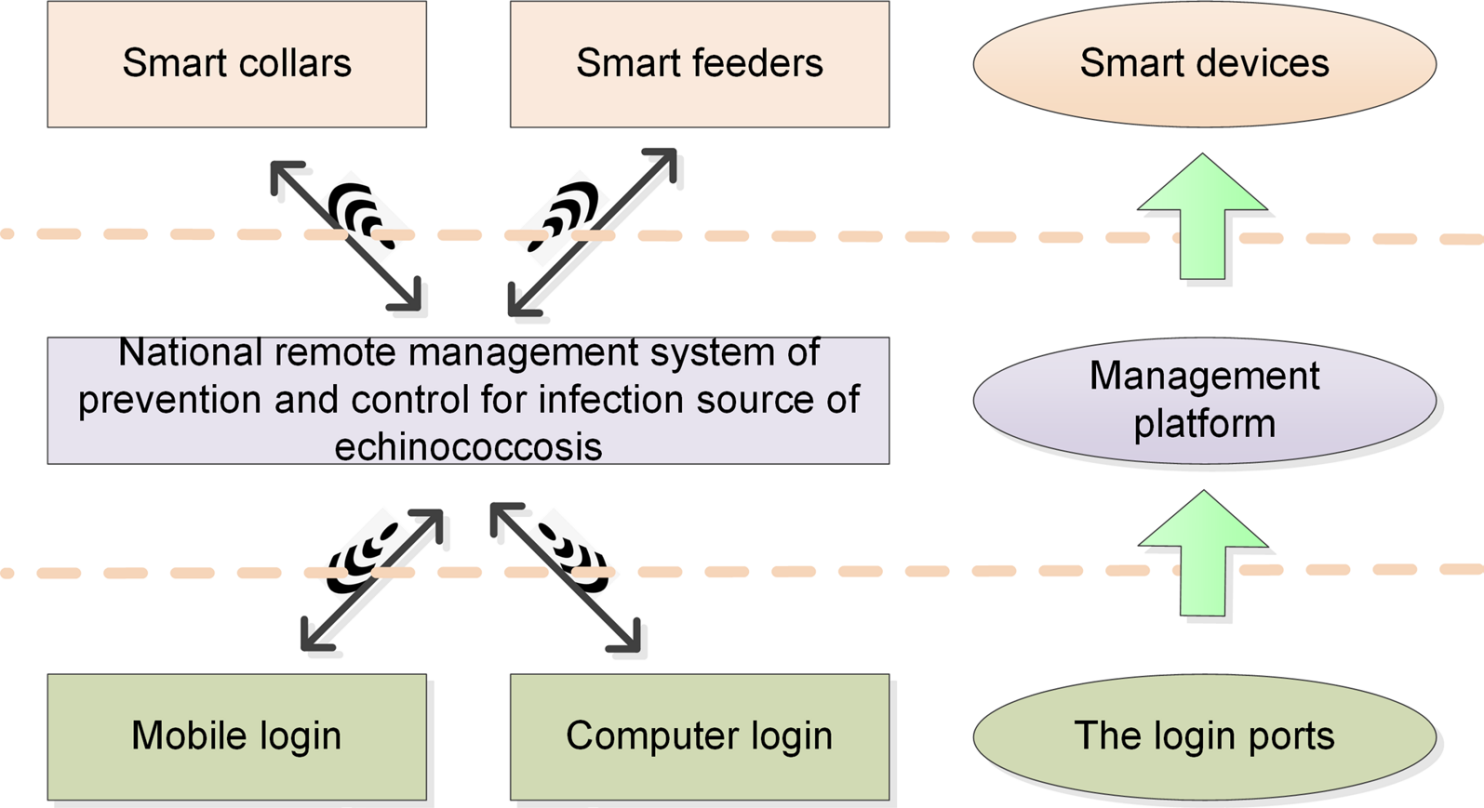
Overall running framework of RMS. RMS: Remote Management System

**Fig. 3:**
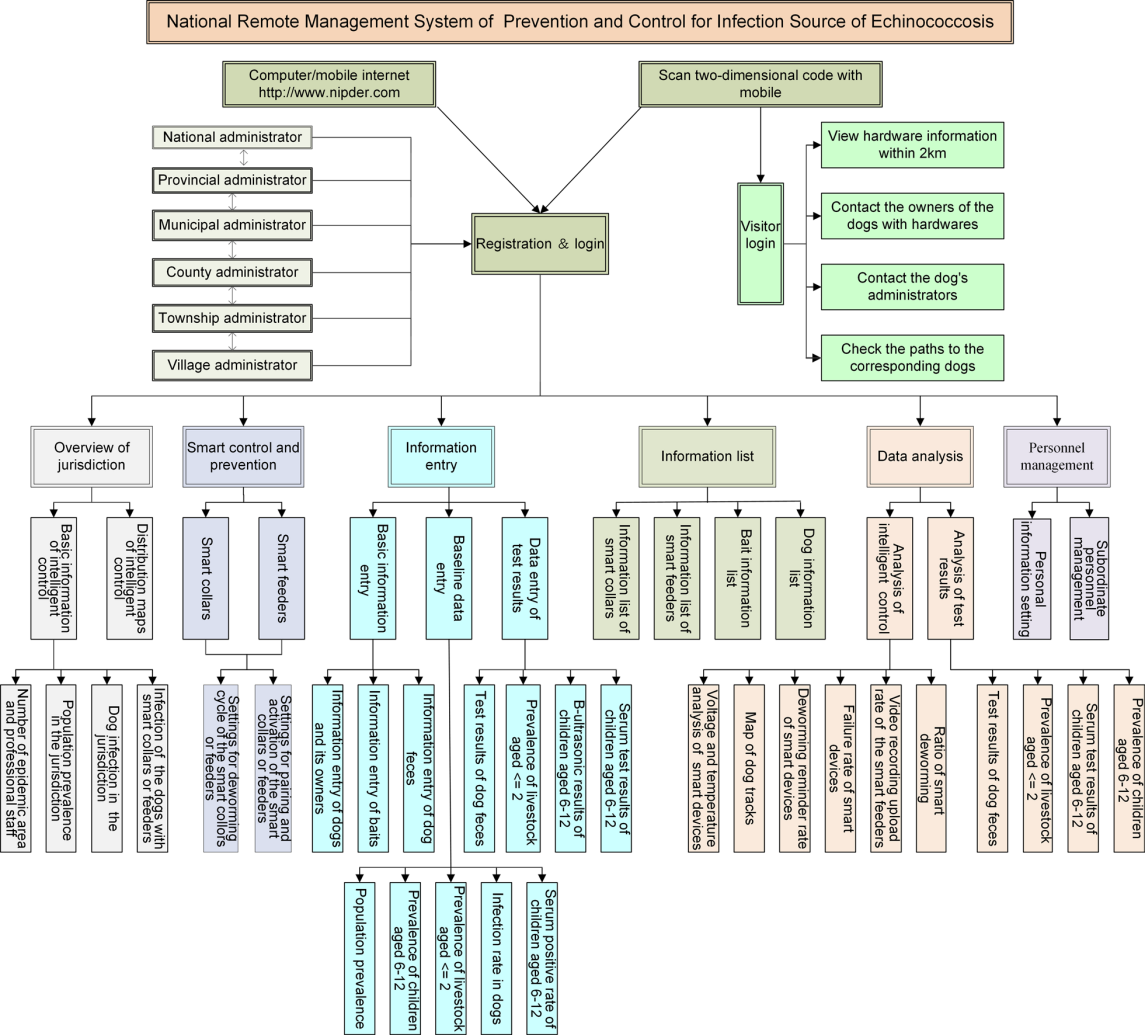
Function architecture of RMS. RMS: Remote Management System

The architecture stack of RMS based on IoT has five layers (Fig. 1). The running framework of RMS includes smart devices, a management platform, and the login port (Fig. 2). The main function of the smart devices (e.g., the collar and the feeder) is to control the definitive host, especially in delivering PZQ baits. The functional display of the management platform has six main components: an overview of jurisdiction, intelligent prevention and control, information input, information display, data statistics, and administrator management (Fig. 3).

### Control for various functions of the smart devices

The smart collars are fully capable of anti-collision, waterproof, and cold-proof performance, and the battery’s energy is sufficient, the anti-collision rate, water-proof rate, cold-proof rate and voltage normal rate is 99.6% (521/523), 100.0% (523/523), 100.0% (523/523) and 100.0% (523/523), respectively (Table 2). The RMS can realise the control of various functions of the smart devices, the automatic delivering PZQ rate, collar positioning rate, deliver PZQ reminding rate, and fault report rate is 91.1% (1914/2102), 92.1% (13 580/14 745), 92.1% (1936/2102) and 84.7% (1287/1519), respectively (Table 3).

**Table 2:** Performance of smart device displayed by remote management system (RMS)

Date	Total number of collars	Water-proof rate (%)	Anti-collision rate (%)	Cold-proof rate (%)	Voltage normal rate (%)
2019.09	20	20/20 (100.0)	20/20 (100.0)	20/20 (100.0)	20/20 (100.0)
2019.10	156	155/156 (100.0)	156/156 (100.0)	156/156 (100.0)	156/156 (100.0)
2019.11	264	264/264 (100.0)	264/264 (100.0)	264/264 (100.0)	264/264 (100.0)
2019.12	523	523/523 (100.0)	523/523 (100.0)	523/523 (100.0)	523/523 (100.0)
2020.01	523	523/523 (100.0)	523/523 (100.0)	523/523 (100.0)	523/523 (100.0)
2020.02	523	522/523 (99.8)	523/523 (100.0)	523/523 (100.0)	523/523 (100.0)
2020.03	523	521/523 (99.6)	523/523 (100.0)	523/523 (100.0)	523/523 (100.0)
Total		521/523 (99.6)	523/523 (100.0)	523/523 (100.0)	523/523 (100.0)

**Table 3:** Functions of smart devices controlled by remote management system (RMS)

Date	Total number of collars	Automatic delivering PZQ rate (%)	Collar positioning rate (%)	Deliver PZQ reminding rate (%)	Fault reportrate (%)	Location upload rate (%)
2019.09	20	20/20 (100.0)	56/60(93.3)^b^	20/20 (100.0)	5/6 (83.3)	20/20 (100.0)
2019.10	156	72/78 (92.3)^a^	1478/1560 (94.7)^b^	73/78 (93.6)	59/67 (88.1)	144/156 (92.3)
2019.11	264	119/132 (90.2)^a^	2468/2640 (93.5)^b^	123/132 (93.2)	121/137 (88.3)	242/264 (91.7)
2019.12	523	285/303 (94.1)^a^	3432/3676 (93.4)^c^	289/303 (95.4)	245/309 (79.3)	477/523 (91.2)
2020.01	523	479/523 (91.6)^a^	1895/2092 (90.6)^d^	491/523 (93.9)	242/284 (85.2)	462/523 (88.3)
2020.02	523	476/523 (91.0)	1869/2092 (89.3)^d^	471/523 (90.1)	278/328 (84.8)	583/523 (92.4)
2020.03	523	463/523 (88.5)	2382/2615 (90.7)^d^	469/523 (89.7)	337/388 (86.9)	488/523 (93.3)
Total		1914/2102 (91.1)	13 580/14 745 (92.1)	1936/2102 (92.1)	1287/1519 (84.7)	496/523 (94.8)

^a^There were 78, 132, 220 smart collars with no deworming time configured, so the PZQ bait was not delivered in September, October and November, respectively; ^b^“once every 3 days” was set as position feedback cycle for each smart collar; ^c^“once every 3 days” set as position feedback cycle for 264 smart collars and “once every 7 days” for 259 smart collars; ^d^“once every 7 days” was set as position feedback cycle for each smart collar

### Data summary and parameters analysis for surveillance

The RMS can accurately summarise and analyse the monitoring data and parameters including positive rates of canine faeces and the prevalence of echinococcosis in the general population, livestock, and children. In smart deworming group, with the increasing of deworming number, the positive rate of dog fecal antigen shows a downward trend (Z = 28.766, *P* < 0.05), however, in manual deworming group, whether it is before or during deworming, the positive samples of dog fecal antigen were always maintained in the same level without significant change (*Z* = 0.107, *P* > 0.05)(Table 4). Before 2019, deworming and management of dogs and surveillance for echinococcosis have always been manual; the data are incomplete, and the data missing rate reached 32.1% (9/28). From 2019, dogs deworming and surveillance for echinococcosis will be controlled using RMS and will be expanded gradually in townships to the whole Hezuo city, and until December 2020, the missing rate of monitoring data decreased to 0 (0/16) (Table 5).

**Table 4:** Change of positive rates of canine faeces controlled by remote management system (RMS)

	Deworming times	Group dewormed by Smart collars	Manual deworming group (%)	*χ*^2^ value	*P* value
Baseline data^8^	/	6/320 (1.88)	/	/	/
Statistical analysis by RMS	0 (Before deworming)	16/474 (3.4)	6/177 (3.4)	0.993	1
	1st	1/475 (0.2)	6/169 (3.6)	12.912	0.002
	2nd	2/487 (0.4)	7/163 (4.3)	10.838	0.004
	3rd	1/467 (0.2)	7/170 (4.1)	15.290	0.001
	4th	0/442 (0)	5/161 (3.1)	13.819	0.001
	5th	0/232 (0)	5/169 (2.9)	6.933	0.013
	6th	0/127 (0)	6/171 (3.5)	4.532	0.04
	7th	0/20 (0)	5/173 (2.9)	0.590	1
*Z* value		28.766	0.107		
*P *value		0.000	0.743		

/ means the data were not be counted

**Table 5:** Completeness of the data of surveillance for echinococcosis by remote management system (RMS) vs manual management

Years	Population prevalence		Prevalence of children		Positive rates of canine faeces		Prevalence of livestock	
	Group A	Group B	Group A	Group B	Group A	Group B	Group A	Group B
2012	9/4006 (0.22)	/	*	/	6/320 (1.88)	/	22/500 (1.7)	/
2013	13/15 000 (0.09)	/	*	/	40/2000 (2.00)	/	*	/
2014	8/10 010 (0.08)	/	*	/	38/2000 (1.90)	/	13/1000(1.30)	/
2015	6/5004 (0.12)	/	*	/	41/2000 (2.05)	/	12/1000(1.20)	/
2016	5/10 000 (0.05)	/	*	/	36/1500 (2.40)	/	10/1000(1.00)	/
2017	2/10 010 (0.02)	/	*	/	32/1500 (2.13)	/	*	/
2018	4/10 046 (0.04)	/	*	/	25/1200 (2.08)	/	7/994 (0.70)	/
2019	4/8546(0.04)	0/300(0)	0/1446(0)	0/554(0)	25/663 (3.77)	4/1871 (0.21)	2/309 (0.65)	1/200 (0.5)
2020								
2021								
2022								

Group A means manual deworming and management for dogs in the township and villages. Group B means smart deworming for dogs in some townships from 2019, and using the RMS to manage the monitoring data of echinococcosis within the county.*means the data were missed. /means the smart deworming and telemanagement had not started. means from 2019 to 2022, the data will be continuous, dynamic, accurate, real-time collected, analyed, and displayed by the RMS

### Using satisfaction rate

A total of 48 administrators (3, 3, 8, 11, 23 at the provincial, municipal, county, township, village levels, respectively) participated in the questionnaire survey. The overall satisfaction rate reached 93.8% (Table 6).

**Table 6:** Results of questionnaire survey on using the remote management system (RMS)

Questions	Number of answers		Res rate, %
	Yes	No	
Is the interface of the RMS friendly?	47	1	97.9
Is the RMS easy to operate?	46	2	95.8
Can various functions be realized?	48	0	100.0
Does the RMS save time and labor?	48	0	100.0
Are you willing to install and use the RMS?	45	3	93.8
Are you satisfied with the RMS?	45	3	93.8

### Operation and results display as a management platform

**Fig. 4:**
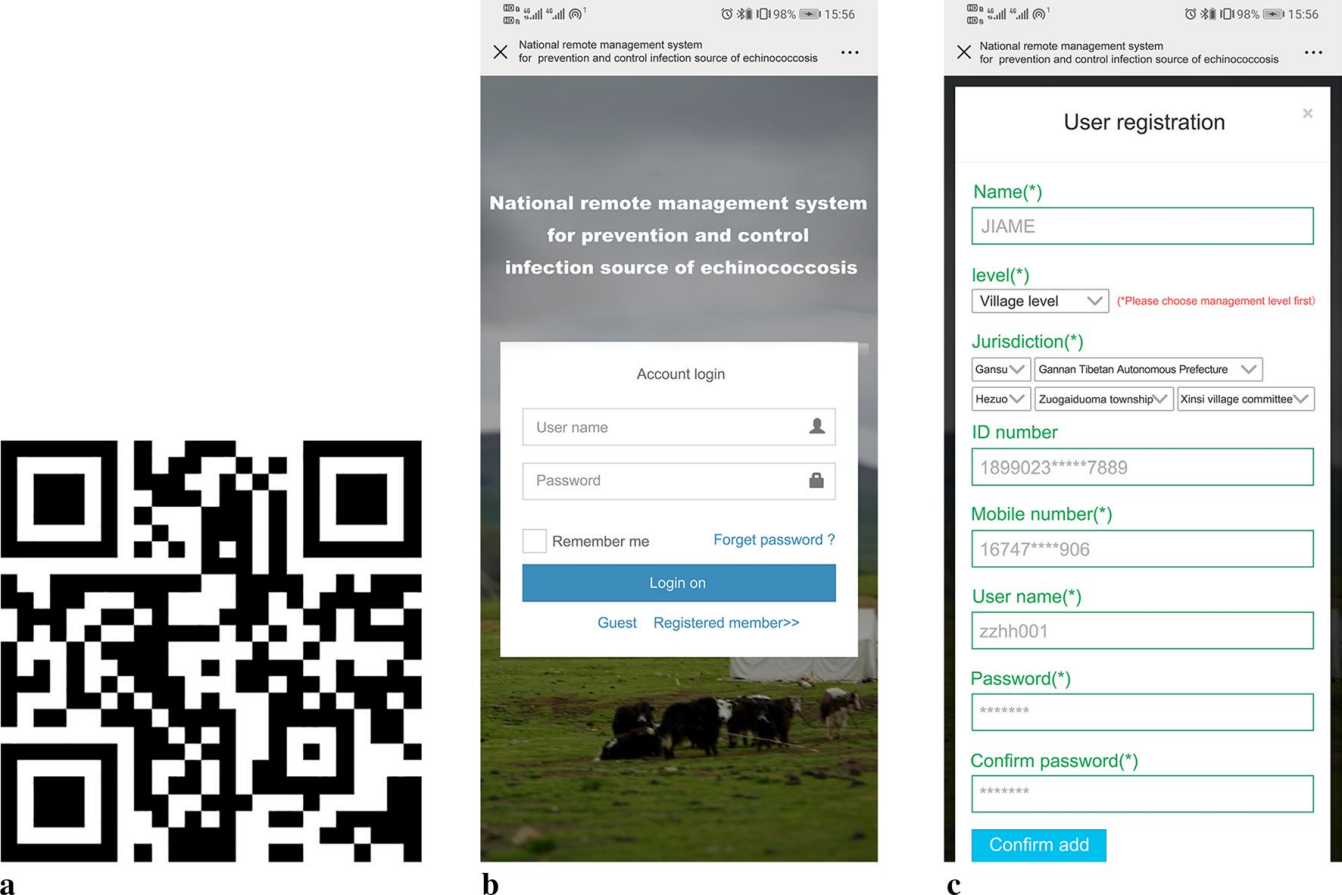
The process of RMS based on IoT (**a** Mobile phone QR code login, **b** Mobile phone registration interface, **c** Mobile phone login interface)

**Fig. 5:**
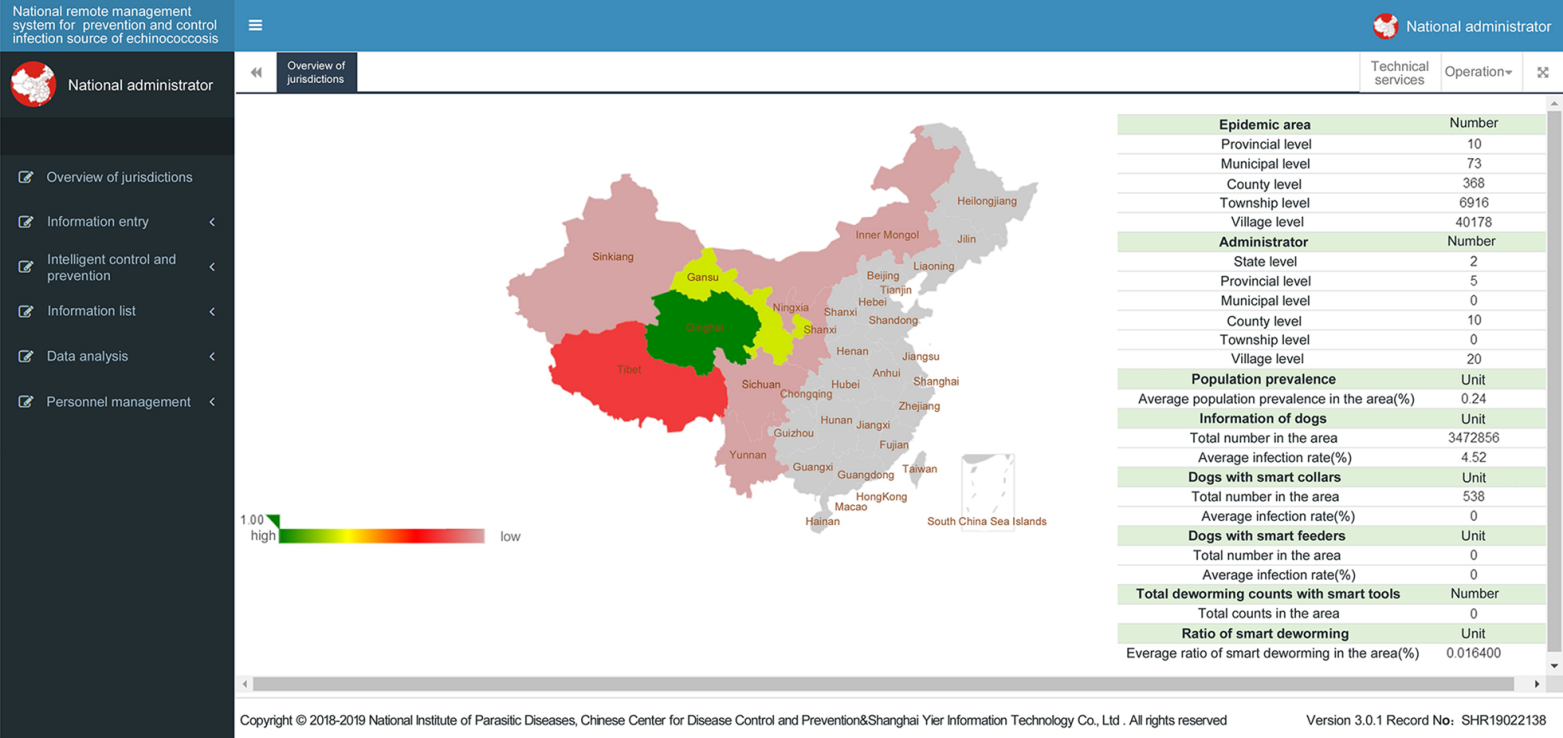
The “overview of the Jurisdiction” interface after the national administrator log-in the RMS

**Fig. 6:**
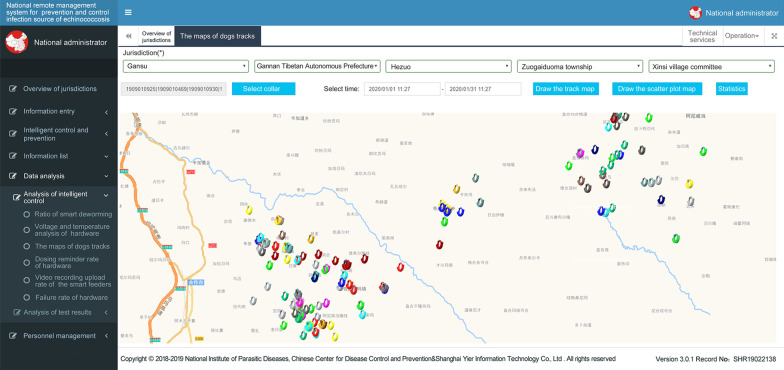
Movement trajectory density map of 523 smart collars in January 1–31, 2020, in Hezuo

The login port comprises the PC terminal, mobile phone web terminal, and WeChat public account. The login URL on the PC and mobile phone web pages is https://www.nipder.com. The interface for registration, login, and activation of administrators in six levels is shown in Figs. 4a–c. The “overview of the jurisdiction” in Fig. 5 displays the basic information of echinococcosis prevention and control in a given jurisdiction. The operation results demonstrated in the movement trajectory density map of 523 smart collars in January 1–31, 2020, in Hezuo (Fig. 6). All the detail results of the RMS operations can be found in the Additional files 1, 2, 3, 4, 5, 6, 7, 8, 9, 10, 11, 12: Figs. S1–12.

## Discussion

In this study, we designed smart deworming devices that use RMS for surveillance of echinococcosis. The steps for control by smart deworming devices are as follows: (1) attach the smart collar on the neck of the target dog or fix the smart feeder near the target dog, (2) match ID of the smart devices with ID of the target dog, owner, the villager, and village administrator, and (3) set deworming frequency, the dosing time, and the deworming dosage in computer or mobile phone. All the information (command feedback) is associated between the RMS and smart devices. Inside the smart deworming device (see Fig. 1), the temperature sensor, GPS sensor, voltage sensor, low-power technology, and timing were employed to ensure the realisation of a series of functions, and the required executive modules of PZQ-delivery, malfunction alarm, video shooting, low-power control, and delivery reminder were all embedded and controlled by the central processing unit(see Fig. 1).The required executive modules of PZQ-delivery(unpublished) includes motor module, delivery module, timing module, power control module, delivery port, and baits storage semicircular. In general, once the smart device is matched and set successfully, the device will be in a sleep state (low power consumption state, only timer and positioning mode work), when 3 min before dosing, the smart device will be activated and emits dosing reminder music. After 3 min, the motor module works, opening delivery port, and ejecting one (or two) PZQ bait(s) from baits storage semicircular, then closes delivery port, and sends relevant information to the RMS, the smart deworming device will enter the dormant state and enter the next deworming cycle. The RMS provides a variety of deworming frequency options, such as once a month, once every two months, once a quarter, and once every six months, which can be selected depending on the local *Echinococcus* infection rates and actual requirements. The dosing date can be set (we set the time as the 30th of each month) and the deworming dosage for each dog can also be set individually (we can set one bait or two baits to be administered to the dog at a time according to the weight of the target dog) on the RMS. Table 4 shows that the positive rate of dog faeces in the manual deworming group is significantly higher than that of the smart deworming group. The smart devices controlled by RMS can greatly reduce the worm biomass in dogs. The “attaching once, lasting a year, deworming 12 times” of the smart deworming devices greatly improves the efficiency and effectiveness of deworming, and saves time, labour, and capital costs, especially in the Tibetan agricultural and pastoral areas with harsh climates. The RMS is a potential replacement of the existing manual deworming and can ensure precise prevention and control of echinococcosis.

The completeness, accuracy, and timeliness of surveillance data are the basic requirements for precise control of echinococcosis. Monitoring includes active mass screening for human echinococcosis, surveillance for canine echinococcosis, and surveillance for echinococcosis in livestock [27]. The main monitoring indicators of the echinococcosis include the positive rates of canine faeces and the prevalence of the disease in the general human population, children, and livestock. The national survey data of echinococcosis from 2012–2016 were incorporated to RMS for real-time dynamic comprehensive analysis on the epidemic status of echinococcosis. Annual surveillance data of echinococcosis in target areas can be added after RMS has been adopted. The comparative analysis of the effectiveness before and after smart deworming can be performed, and the parallel comparative evaluation of the effectiveness of the smart deworming versus manual deworming can also be conducted. Dynamic surveillance can guide decision-making for echinococcosis control and inform the public and the policymakers about the progress and effectiveness of control initiatives. In addition, all the data and results on the RMS can be printed online or exported for further statistical analysis. The RMS can promote the scientific and electronic management, storage, mining, and utilisation of surveillance data, reduce the labour costs and errors, and improve the completeness, accuracy, and effectiveness of the data. The latest version of the RMS has updated the monitoring indicators to be compatible with the upgraded National Hydatid Disease Surveillance Program (Chinese Center for Disease Control and Prevention, Notice of the National Hydatid Surveillance Program, 2020 version).

As a telemanagement platform, friendly and easy to operate interface can greatly improve user experience. The preliminary interviews with administrators at the five levels show that the RMS is both friendly and easy to conduct, and the satisfaction rate reached 93.8%. The content displayed on the interface of the RMS mainly includes overview of the jurisdiction, information entry, information list, smart control and prevention, data analysis, and personnel management. The “overview of the jurisdiction” displays the basic information of echinococcosis prevention and control in a particular jurisdiction including the endemic map embedded in the centre of interface, basic information such as the number of administrators in this jurisdiction and the lower levels, and the number of endemic provinces, cities, counties, townships, and villages. The human population prevalence, the total number of dogs and infection rates, the number of dogs deworming by smart devices, and the infection rates are displayed on the right interface. These data are automatically generated and summarised from the lower-level data. The entire interface is intuitive and visual, and the data is relayed in real time (Fig. 5). Information entry mainly includes the input of basic information, baseline data, and test results (Fig. 3). Information lists enable the administrators to browse and query related information in real time. Smart control and prevention enable the administrators to browse and query the operating status of smart deworming devices as well as fault repair and teleguidance. The interface of data analysis has focused not only on smart control data and monitoring data but also on the movement trajectory of each dog over a particular period. The smart collars or feeders help the RMS to trace the dogs to a village, a township, or even a county. Therefore, it is possible to understand the precise movement scope of the infection source of echinococcosis and implement targeted preventive control measures. In addition, the interface of voltage and temperature can help the administrators know the actual status of the smart devices in real time and determine whether batteries of the given collar need to be replaced or recharged.

During the development process of the RMS from 2016 to 2019, a worrisome fundamental concern was the availability of wireless network coverage in the echinococcosis-endemic area of alpine Tibetan. Without reliable wireless network coverage, the RMS would not achieve the goal of remote control and surveillance for echinococcosis. It is gratifying that China’s rapid economic development over the past few decades, especially driven by China's Western Development Policy and the rapid improvement of mobile communication technology, has provided a strong network foundation and feasibility for the RMS. In 2019, in the Tibet Autonomous Region, the penetration rate of fixed broadband reached 98.7 per 100 households, the broadband coverage of the administrative villages reached 100.0%, the coverage rate of the 3G network reached 100%, the administrative village realised full coverage of mobile signals [28]. In Gansu, the mobile phone penetration rate is 104.3 units/100 people, and the penetration rate of mobile broadband access users is 90.8 per 100 people[29]. Therefore, the internet or wireless network and telecommunication coverage do not seem to be a serious problem in the echinococcosis-endemic area of Qinghai-Tibet Plateau. In this study, 94.8% of smart collars could successfully connect to the local wireless network and upload the related information to RMS. But even so, to ensure that the coverage and the frequency of deworming for dogs are not affected by the wireless network, a self-control loop and data storage unit were embedded in the smart devices. They guarantee that, even if there is no wireless network, the smart deworming devices can automatically deliver PZQ baits regularly and, quantitatively and record the information about delivery reminders, fault alarms, location, working temperature, and battery voltage and save them in the CPU. Once the wireless network is restored, the smart devices can connect to the RMS and upload the stored information automatically.

Considering that the smart deworming devices controlled by RMS will be employed in scattered nomadic communities with low socio-economic status, we hope to provide a smart comprehensive solution with affordable cost to replace the existing manual management model. According to the company's quotation, the maintenance fees for RMS (rental fees) is about 0.3 USD/month/per set, the smart deworming collar is about 35 USD / per set/ year, the smart deworming feeder is about 50 USD/ per set/year, the service period of the smart devices (free maintenance and replacement if damaged unintentionally) from the manufacturer is three years. Of course, the actual purchase price will be reduced as the scope of application expands, the using numbers increases, and the production technology capacity increases. In general, this cost is acceptable compared with the existing manual deworming management model (this model includes establishing stray dog shelters, killing stray dogs, deworming domestic dogs, dog sterilization, etc.).

Although the RMS has a reasonable architecture, stable performance, strong operability, and friendly interface, there is a need to improve several limitations as follows. (1) The interfaces and their related content should be further optimised. For example, the upgraded “Overview of Jurisdiction” should increase to display the dynamic change graphs, including the population prevalence, the infection rate of dogs, the prevalence of children, and the prevalence of livestock in a particular jurisdiction; the training contents (i.e. audiovisual or animation) for application personnel at different levels should also be embedded in the corresponding interface; the content of disease transmission education for the most basic personnel also should be added in order to improve the consciousness of performing the required tasks; (2) The statistical analysis should be enriched by adding a variety of statistical analysis methods, even drawing and generating two-dimensional or three-dimensional charts, and linking it to relevant statistical analysis software to establish an early warning module for the echinococcosis epidemic; (3) Accordingly, there is a need to adjust and optimise the database tables, contents, and evaluation indicators to give the RMS a more highlighted focus, smoother running, and more reasonable layout; (4) Some new functional modules such as data backup module, system optimisation, and update module should also be established;(5) The Huawei cloud server will be used to ensure that the application environment is reliable, flexible, efficient, and safe for the RMS. Now, the RMS has been upgraded and iterated three times up to Version 3.0.1 (Record No: SHR19022138) and has been tested and evaluated in Hezuo since 2019 with no serious adverse events such as crash, data disorder, and information divulgence has been recorded. Moreover, it has passed the stress and load tests and obtained the network licence issued by the industry competent department.

## Conclusions

The RMS offers fast network transmission, timely response, and high stability during application, and the human–machine interface is simple and friendly. The RMS has realised a series of functions, including remote control of delivering PZQ baits regularly and automatically and the statistical analysis and result display of the data. We hope the RMS will drive a paradigm shift of the control and surveillance of echinococcosis from the existing manual implementation to smart, automatic, and precise telemanagement. The existing difficulties and challenges in the way of prevention and control for echinococcosis can partially be resolved using the innovative, IoT-based technologies and tools. The proposed RMS to advance the upgrade of existing manual prevention and control models for echinococcosis, especially in the current ongoing COVID-19 pandemic, as social distance and community blockade continue.

## Supplementary Information

**Additional file 1: Figure S1.** The input interface of the dog and the owner registration and information.

**Additional file 2: Figure S2.** The input interface of PZQ bait information.

**Additional file 3: Figure S3.** The input interface of dog faeces collection information.

**Additional file 4: Figure S4.** Input and user-interface of the population prevalence of baseline data in Gansu Province.

**Additional file 5: Figure S5.** The entry process and user-interface of the prevalence of children in Gansu Province.

**Additional file 6: Figure S6.** Interfaces of smart collar configuration, activation and administration cycle setting.

**Additional file 7: Figure S7.** The interface of information list for dogs.

**Additional file 8: Figure S8.**The interface of information list for smart collars.

**Additional file 9: Figure S9.** Interface for statistics list of smart deworming ratio.

**Additional file 10: Figure S10.** Voltage and temperature changes of the smart collar from Sep 23, 2019 to Feb 3, 2020, in Hezuo.

**Additional file 11: Figure S11.** The analysis interface of deworming reminder rate and failure report rate.

**Additional file 12: Figure S12.** Infection status interface of smart deworming dogs in Hezuo, Gansu province.

## Data Availability

Not applicable.

## Abbreviations

RMS: Remote Management System
IoT: Internet of Things
PZQ: Praziquantel
COVID-2019: Coronavirus Disease 2019
NHC: National Health Commission
WHO: World Health Organization
OIE: World Organization for Animal Health
CDC: Centre for Disease Control and Prevention
PC: Personal computer
CPU: Central processing unit
TCP: Transmission control protocol
