# Supplementary figures and images for: A remote management system for control and surveillance of echinococcosis: design and implementation based on internet of things
Source: Infect Dis Poverty. 2021 Apr 13;10:50. doi: 10.1186/s40249-021-00833-4 (PMC8042360; doi:10.1186/s40249-021-00833-4)

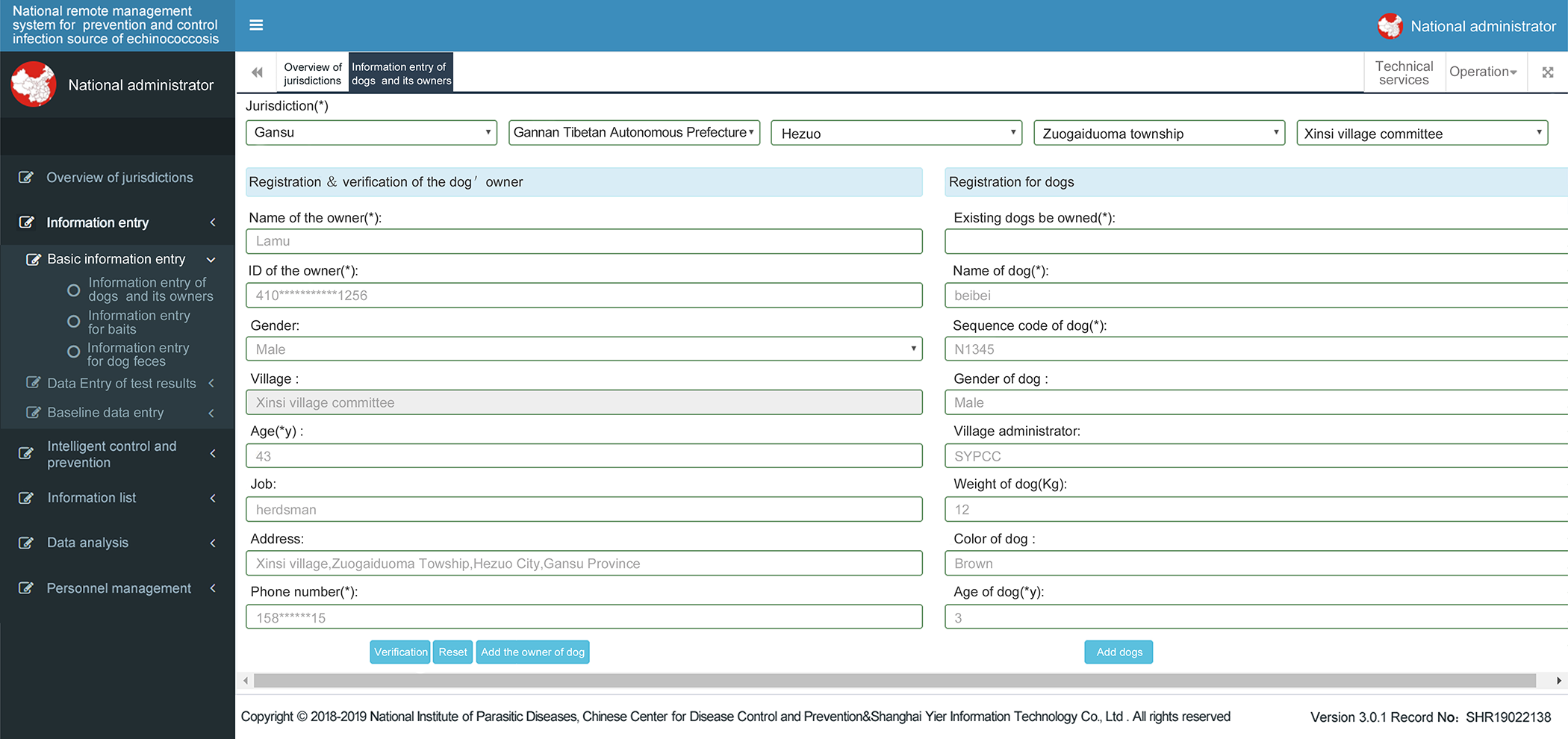

Supplement: Supplementary file 1 — Additional file 1: Figure S1. The input interface of the dog and the owner registration and information. [file 40249_2021_833_MOESM1_ESM.tif]

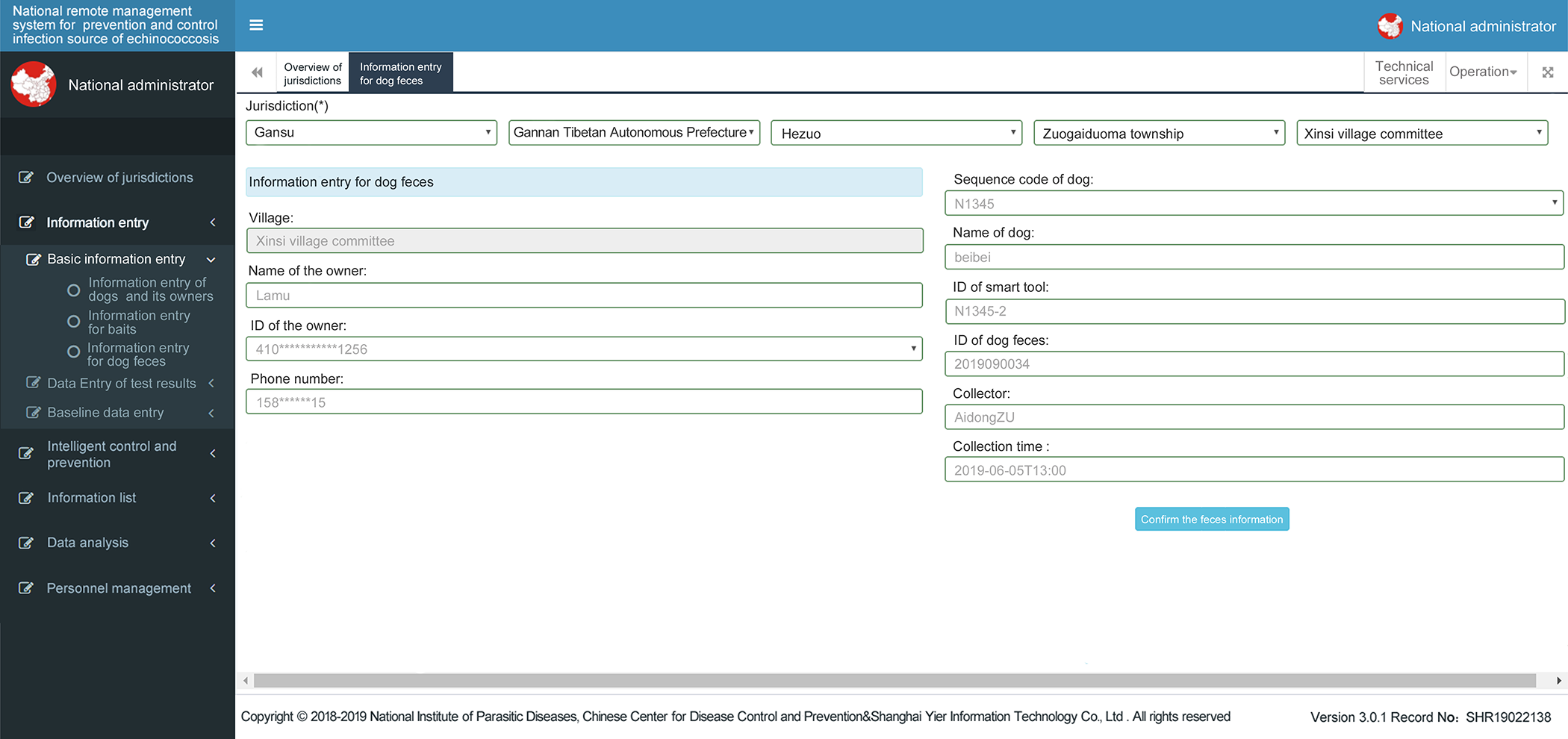

Supplement: Supplementary file 2 — Additional file 2: Figure S2. The input interface of PZQ bait information. [file 40249_2021_833_MOESM2_ESM.tif]

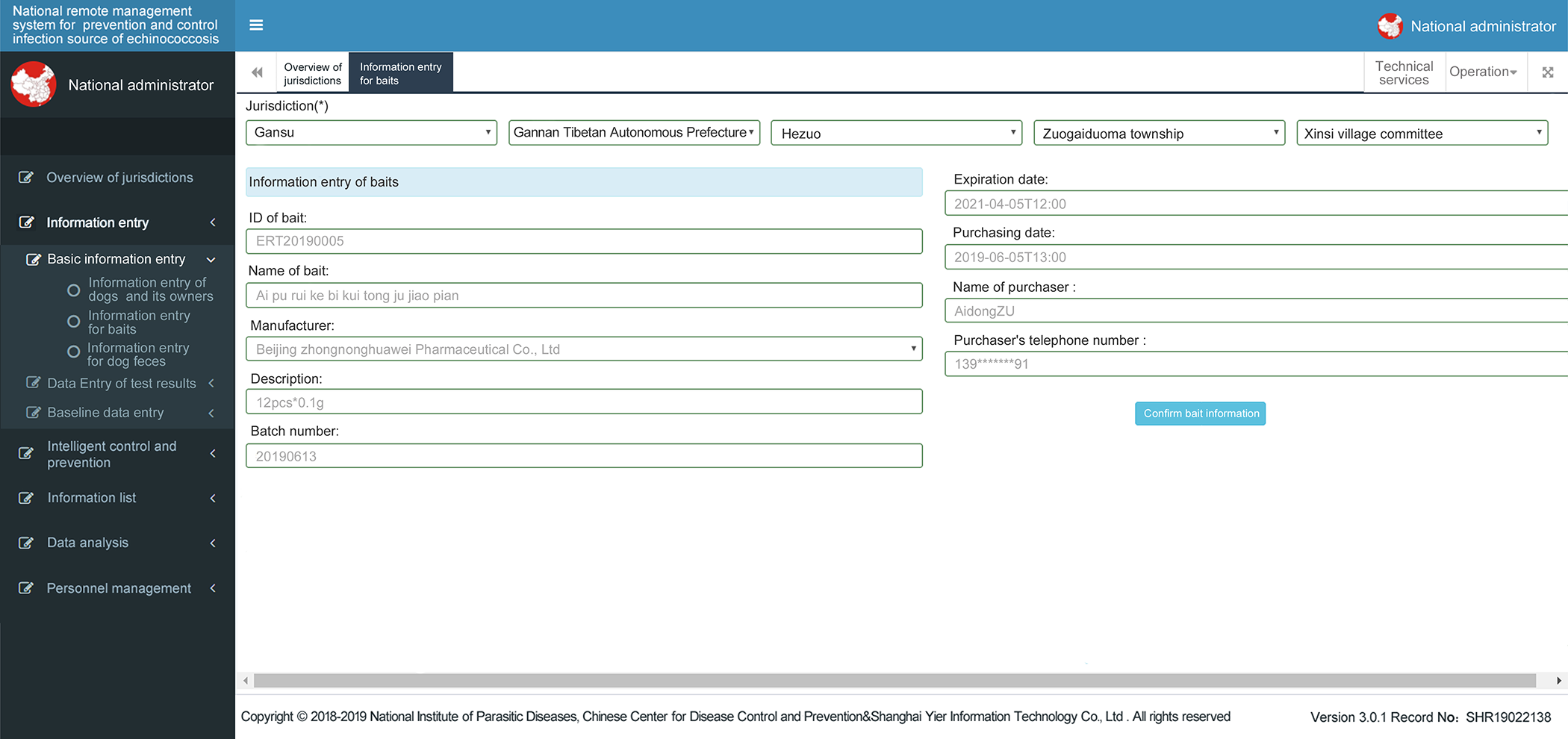

Supplement: Supplementary file 3 — Additional file 3: Figure S3. The input interface of dog faeces collection information. [file 40249_2021_833_MOESM3_ESM.tif]

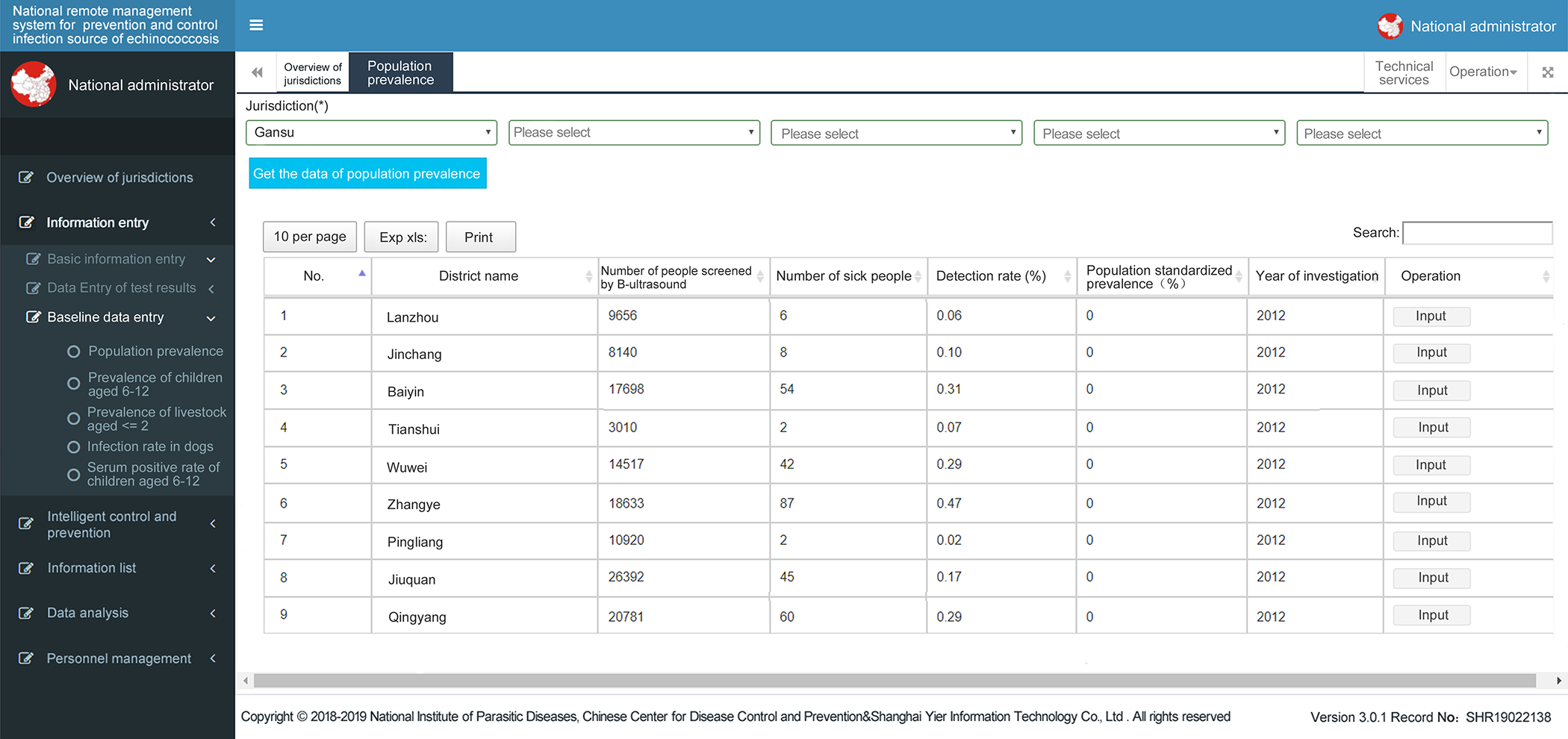

Supplement: Supplementary file 4 — Additional file 4: Figure S4. Input and user-interface of the population prevalence of baseline data in Gansu Province. [file 40249_2021_833_MOESM4_ESM.tif]

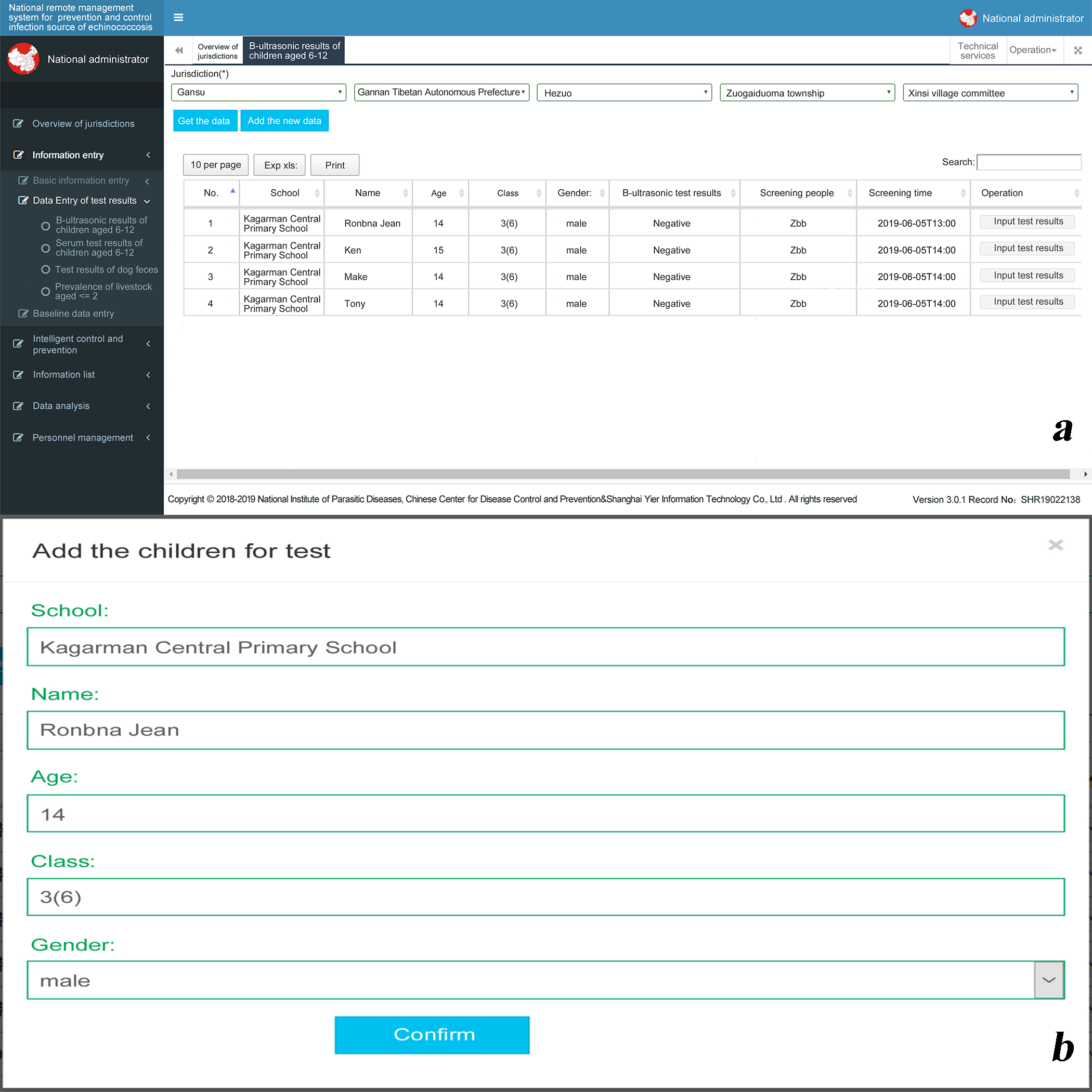

Supplement: Supplementary file 5 — Additional file 5: Figure S5. The entry process and user-interface of the prevalence of children in Gansu Province. [file 40249_2021_833_MOESM5_ESM.tif]

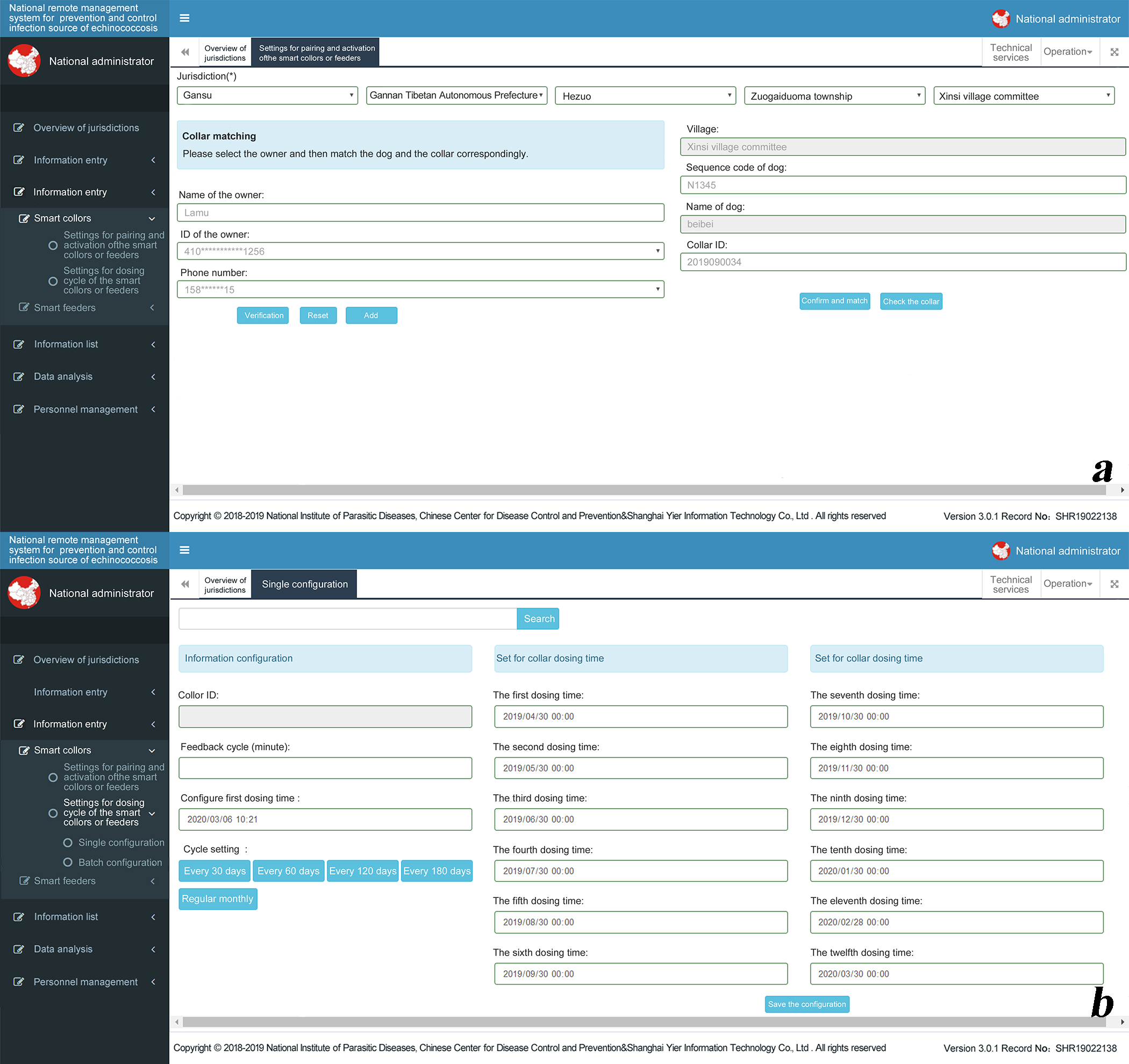

Supplement: Supplementary file 6 — Additional file 6: Figure S6. Interfaces of smart collar configuration, activation and administration cycle setting. [file 40249_2021_833_MOESM6_ESM.tif]

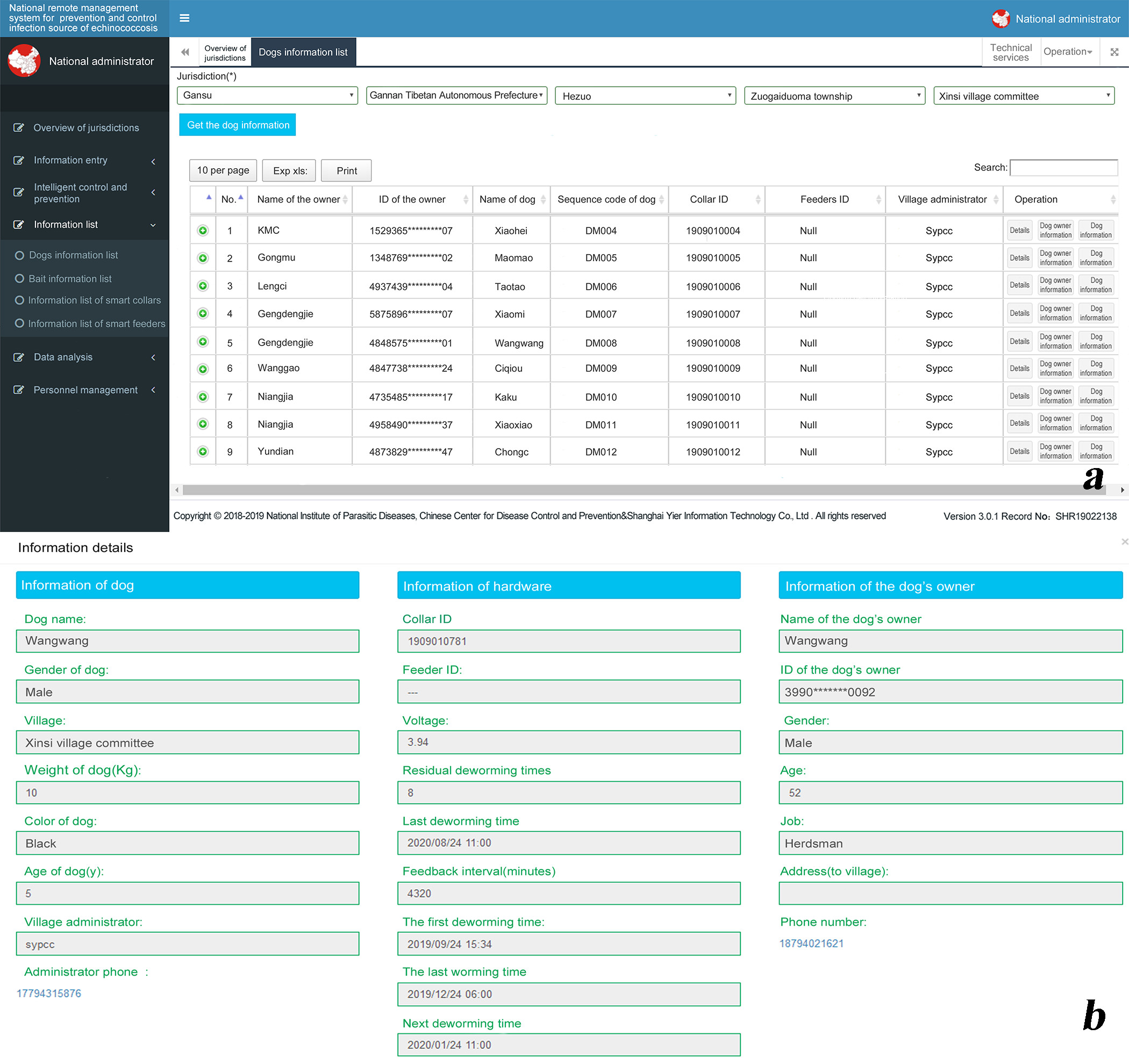

Supplement: Supplementary file 7 — Additional file 7: Figure S7. The interface of information list for dogs. [file 40249_2021_833_MOESM7_ESM.tif]

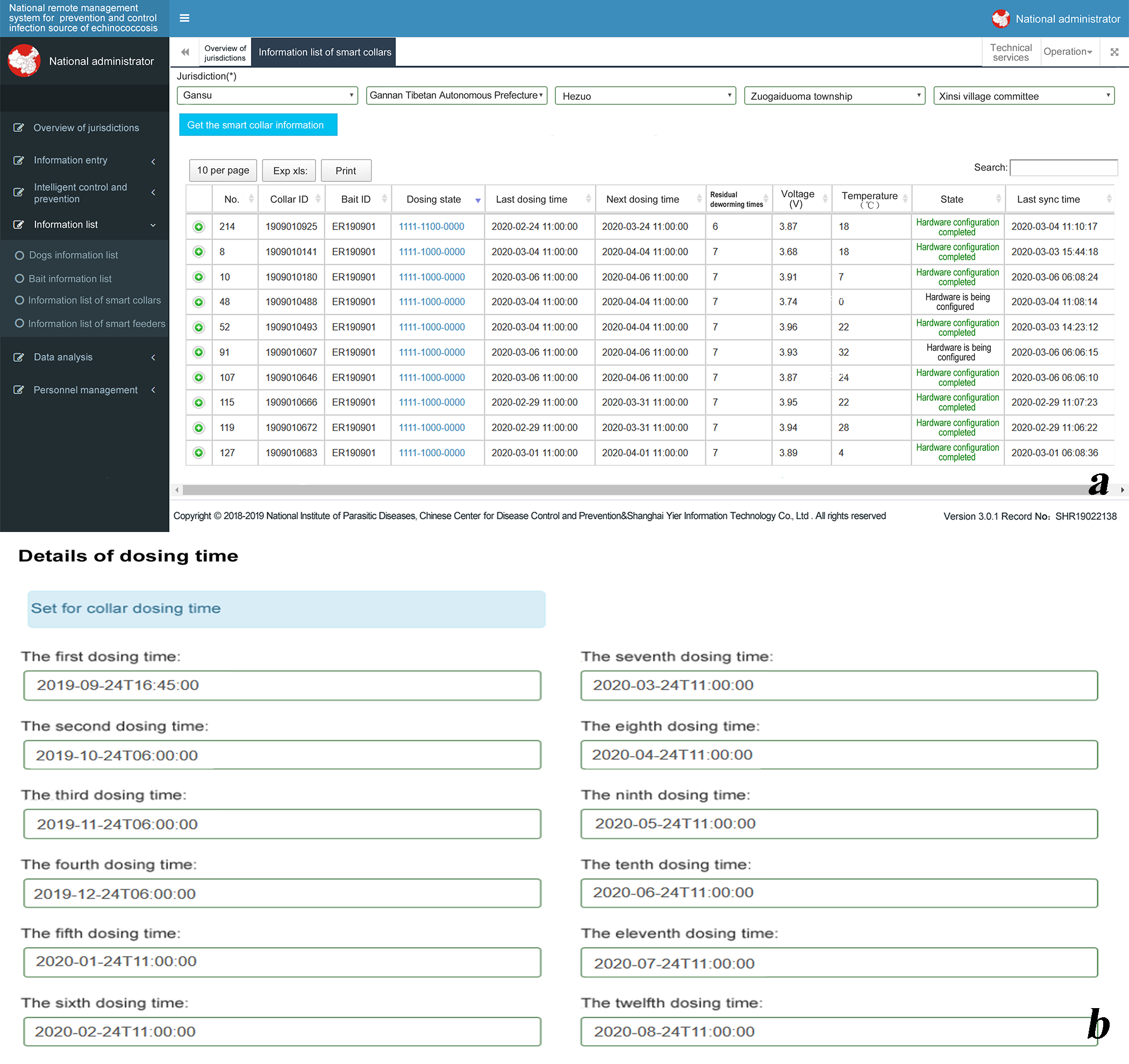

Supplement: Supplementary file 8 — Additional file 8: Figure S8.The interface of information list for smart collars. [file 40249_2021_833_MOESM8_ESM.tif]

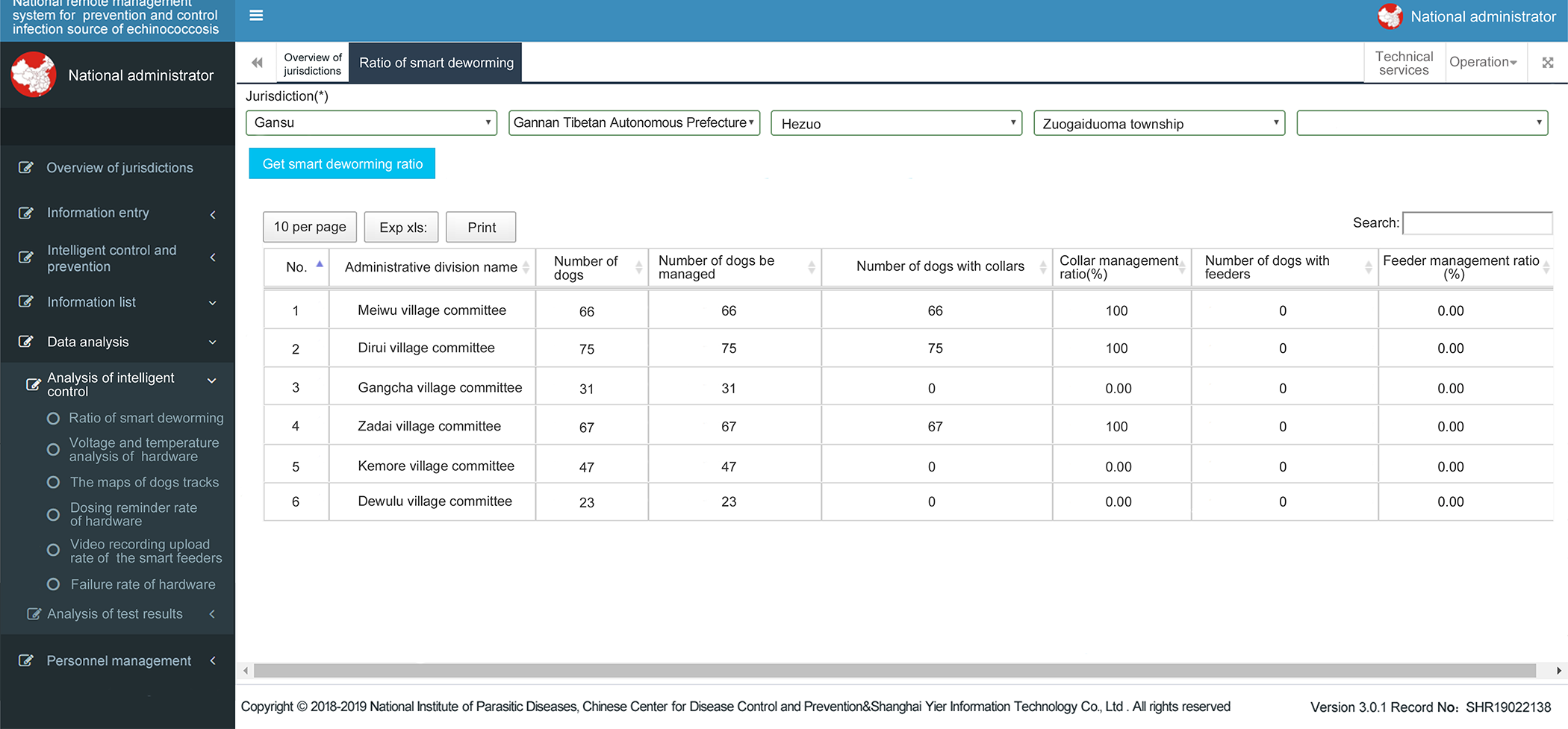

Supplement: Supplementary file 9 — Additional file 9: Figure S9. Interface for statistics list of smart deworming ratio. [file 40249_2021_833_MOESM9_ESM.tif]

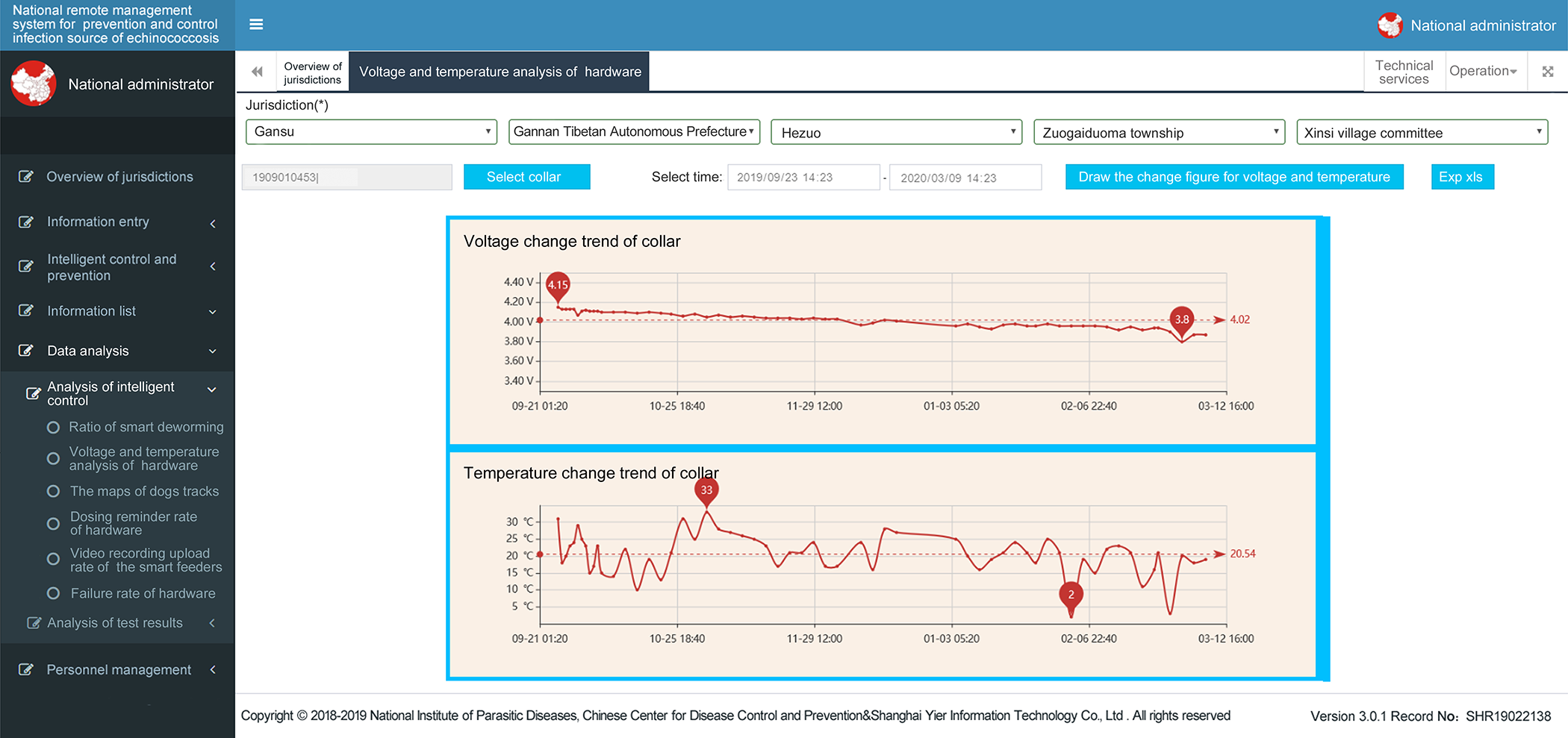

Supplement: Supplementary file 10 — Additional file 10: Figure S10. Voltage and temperature changes of the smart collar from Sep 23, 2019 to Feb 3, 2020, in Hezuo. [file 40249_2021_833_MOESM10_ESM.tif]

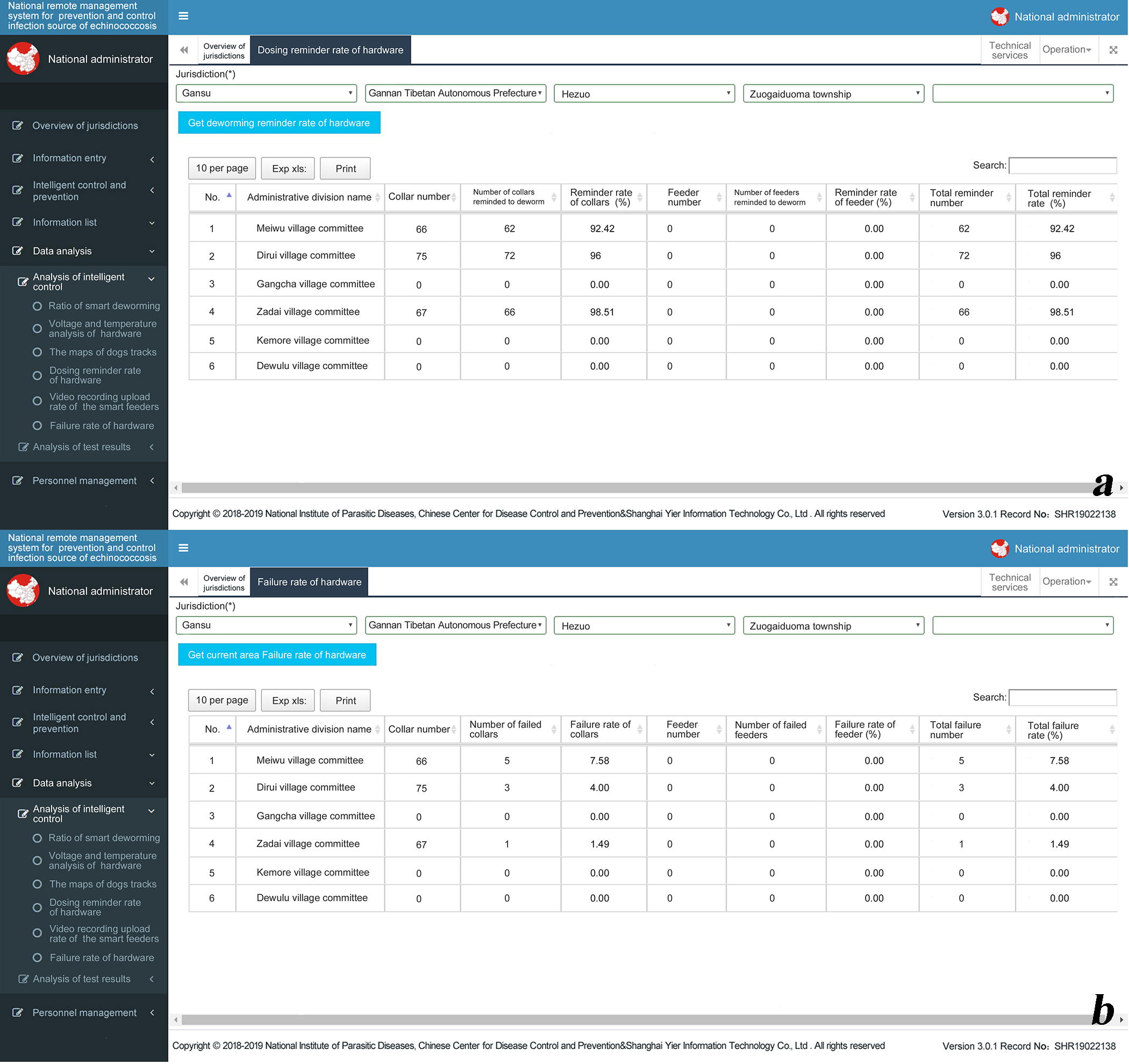

Supplement: Supplementary file 11 — Additional file 11: Figure S11. The analysis interface of deworming reminder rate and failure report rate. [file 40249_2021_833_MOESM11_ESM.tif]

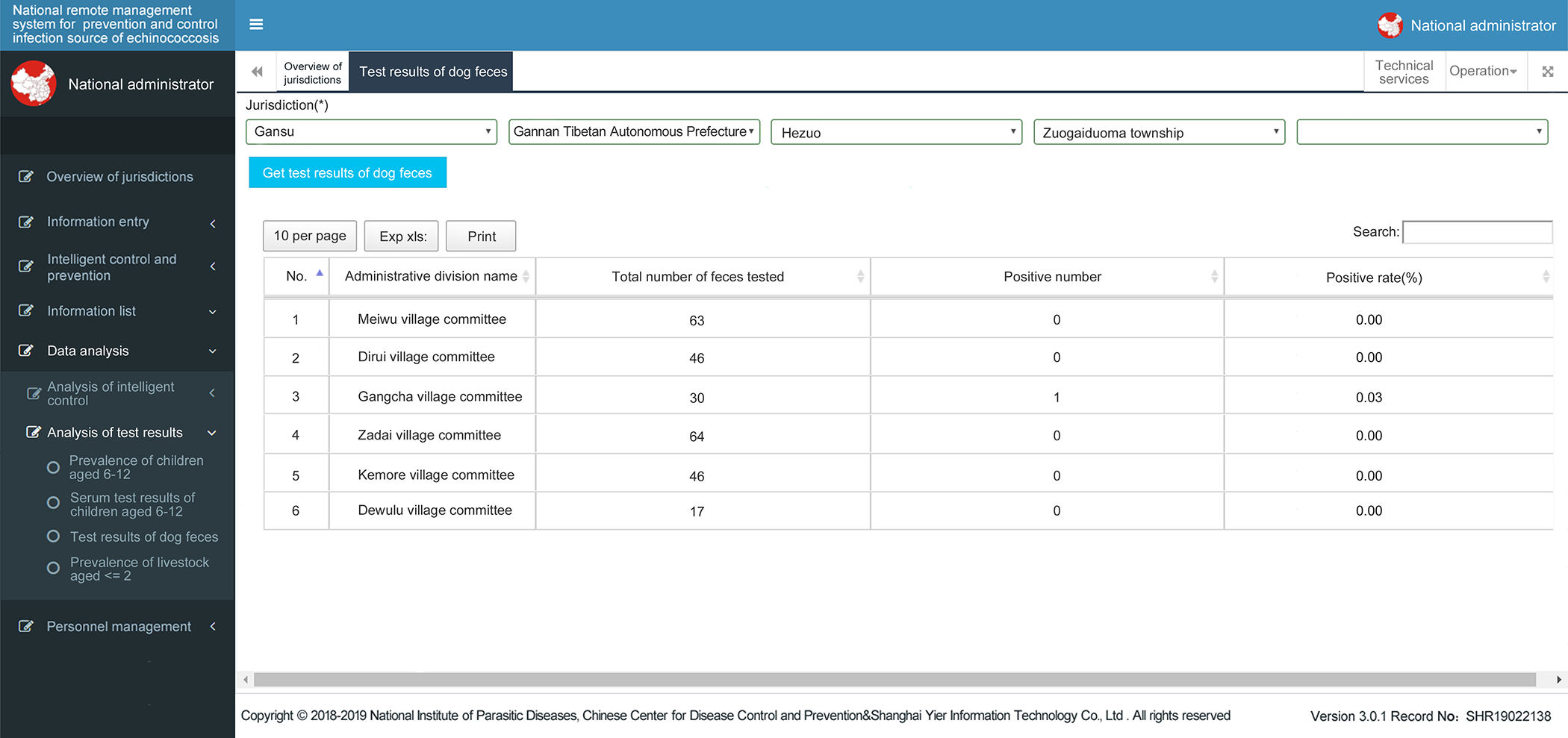

Supplement: Supplementary file 12 — Additional file 12: Figure S12. Infection status interface of smart deworming dogs in Hezuo, Gansu province. [file 40249_2021_833_MOESM12_ESM.tif]
